# Food insecurity among families with infants born during the COVID-19 pandemic in Fortaleza, Northeast Brazil

**DOI:** 10.1186/s41043-023-00354-w

**Published:** 2023-03-05

**Authors:** Simone Farías-Antúnez, Márcia Maria Tavares Machado, Luciano Lima Correia, Hermano Alexandre Lima Rocha, David Augusto Batista Sá Araújo, Maria Suelly Nogueira Pinheiro, Camila Machado Aquino, Ana Luiza Penna, Marcia C. Castro

**Affiliations:** 1 Department of Health Science, Federal University of Santa Catarina, 3201 Governador Jorge Lacerda Street, Araranguá, SC 88906-072 Brazil; 2 Department of Community Health, Federal University of Ceará, 1223 Papi Junior Street, Fortaleza, CE 60430-235 Brazil; 3 Department of Maternal and Child Health, Federal University of Ceará, Fortaleza, Brazil; 4 Department of Global Health and Population, Harvard T. H. Chan School of Public Health, 665 Huntington Ave, Boston, MA 02115 USA

**Keywords:** Food insecurity, Child health, COVID-19, Cohort study

## Abstract

**Purpose:**

To assess the prevalence of food insecurity (FI) among families with infants born during the COVID-19 pandemic and its associated factors in Fortaleza, the fifth largest city in Brazil.

**Methods:**

Data from two survey rounds of the Iracema-COVID cohort study collected at 12 (*n* = 325) and 18 months (*n* = 331) after birth. FI was measured using the Brazilian Household Food Insecurity Scale. FI levels were described according to potential predictors. Crude and adjusted logistic regressions with robust variance were used to assess factors associated with FI.

**Results:**

In the 12- and 18-month follow-ups interviews, there was a 66.5% and 57.1% prevalence of FI, respectively. Over the study period, 3.5% of the families persisted in severe FI and 27.4% in mild/moderate FI. Households headed by mothers, with more children, low education and income, sustained maternal common mental disorders, and that were beneficiaries of cash transfer programs were the most affected by persistent FI.

**Conclusions:**

Although the prevalence of FI decreased in our sample, almost 60% of families in Fortaleza still have no regular access to enough and/or nutritionally appropriate food. We have identified the groups at higher FI risk, which can guide governmental policies.

**Supplementary Information:**

The online version contains supplementary material available at 10.1186/s41043-023-00354-w.

## Introduction

Food insecurity (FI) is defined as the lack of regular access to enough, safe, and nutritious food necessary for normal growth and development and for having an active and healthy life. It may happen due to the unavailability of food and/or lack of resources to obtain food [13]. Living with FI affects short- and long-term health outcomes. Food insecurity has been associated with a higher risk of poor health, worse emotional well-being, worse school engagement among children, and limitations in activities of daily living among seniors [2, 17]. Living with FI is especially critical for children under the age of two, as it can lead to developmental delays [12].

It is estimated that between 702 and 828 million people faced hunger in 2021 worldwide (corresponding to about 10% of the world population), 46 million more than in 2020, and 150 million more than in 2019, before de the COVID-19 pandemic [14].

Brazil experienced chronic hunger in the 1990s, with over a quarter (27.4%) of the population living below the poverty line, most in the North and Northeast regions [29]. In 2003, Brazil launched two governmental programs that contributed to a decline in hunger, the Zero Hunger Program and the conditional cash transfer *Bolsa Família* [28, 33, 37]. The percentage of households with FI declined from 44.9% in 2004 to 22.6% in 2013 [19]. This trend, however, suffered a setback in 2015, when an economic crisis started and the Gross Domestic Product (GDP) declined by 3.5% [20]. The prevalence of FI increased to 36.7% in 2017–18, with 5% of those facing hunger [19].

The COVID-19 pandemic exacerbated the crisis. Brazil ranks second in the number of deaths due to COVID-19 in the world [42]. The GDP declined by 4.1% in 2020 [20], and the economic growth in the 2011–2020 decennial was the worst in the last 120 years [3]. The accumulated inflation in 2021 was 11.4% for the lower-income strata compared to 9.3% for those in the highest [23], highlighting the growth in inequities during the period. As for FI, 55.2% and 58.7% faced some form of FI in 2020 and 2021, respectively [34, 35]. In 2020, 19.1 million Brazilians suffered hunger; in 2021, this number increased to 33.1 million [34, 35]. There is a social gradient in FI and hunger, with the highest burden among households whose head is a woman, those with children under 10 years of age, and those that receive some form of cash transfer benefit. There is also a regional gradient. The North region had the highest FI level in 2017–8 (57.0%) followed by the Northeast (50.3%) [19]. In 2020, during the COVID-19 pandemic, food insecurity affected over 60% of the households in the North and 70% in the Northeast of Brazil with nearly 7.7 million people facing severe hunger in the Northeast [35].

To the best of our knowledge, few cohort studies evaluated food insecurity in families with infants living in the developing world during the pandemic. Here, we leverage data from a birth cohort study (Iracema-COVID) that followed women in a capital city in the Northeast region (Fortaleza, the fifth largest city in Brazil), who were pregnant during the COVID-19 pandemic (including a period of strict lockdown) and delivered in July and August of 2020. Those women faced social, economic, and health challenges that could compromise pregnancy, delivery, maternal health, and child health and development. Our goal is to assess FI, as well as its associated factors, among this population.

## Materials and methods

Fortaleza is the capital city of the state of Ceará located in the Northeast region of Brazil, with an estimated population of approximately 2.7 million inhabitants in 2020, and a Human Development Index (HDI) of 0.754 in 2010 [21]. Between March 15 and May 5, 2020, the city went through a strict lockdown as a measure to contain the spread of COVID-19. Food insecurity was 46.9% in Ceará in 2018, the eleventh highest in Brazil [22].

A cohort study launched in 2020, called Iracema-COVID [10], that included 351 mother–child dyads, facilitates assessing FI in families with children born during the COVID-19 pandemic. Here, we used data from two rounds of the Iracema-COVID cohort study. The study was approved by the National Research Ethics Committee in Brazil (number 73516417.4.0000.5049). Informed verbal consent was obtained from all participants.

Iracema-COVID was designed to evaluate the health status of women who were pregnant during the lockdown period and who delivered in July or August 2020. Four survey rounds have been conducted (at 6, 12, 18, and 24 months after birth). Here, we used information from the second (12 months, 92.9% follow-up rate) and third (18 months, 94.6% follow-up rate) rounds. No food security data were collected at 6 months. Twelve trained interviewers contacted the families at each round and performed home interviews. Rounds 2 and 3 were conducted between July and October 2021 and between February and March 2022, respectively. The detailed design of the survey is described elsewhere [10, 26].

The questionnaires applied in rounds 2 and 3 included the Brazilian Household Food Insecurity Measurement Scale (EBIA), a 14-items scale validated to assess FI perception [38, 39]. Each affirmative answer for any of the 14 items is assigned one point and “no” and “do not know” answers get zero points, generating a score ranging from 0 to 14 points. This score enables the estimation of food security (FS) prevalence (0 points) and FI (≥ 1 point), classifying households of children under 18 years of age into three levels of FI severity: mild (1–5 points), moderate (6–9 points), and severe (10–4 points). We analyzed the prevalence of FS and FI in each survey round, as well as transitions across rounds.

Possible factors associated with FI were identified through a review of the current literature [25, 30] and are described in Additional file 1: Table S1. Socioeconomic status was assessed based on the Brazilian Economic Classification Criteria (CCEB) [1]. The index is summarized into five socioeconomic strata: A (monthly income of US$ 4,049.49), B1 (US$ 1,922.35), B2 (US$ 1,067.76), C1 (US$ 607.93), C2 (US$ 364.73), and D/E (US$ 167.09) (monthly income shown in US dollars calculated using the exchange rate of July 5, 2022, 1 U.S. dollar = 5.39 Brazilian Reais) [1]. Maternal working arrangements were classified into informal and formal according to Brazilian legislation [5]. Maternal common mental disorders (CMD) were assessed by the Self Report Questionnaire (SRQ-20), a 20-item self-report screening tool developed by the World Health Organization (WHO) aimed to detect psychological distress [4]. The SRQ-20 was validated for application to the Brazilian population using a cut-off point of eight or more as an indicator of morbidity with an 83% sensitivity and 80% specificity [27]. Breastfeeding (BF) patterns at 6 months were categorized as exclusive or predominant, complementary, and bottle feeding [15], World Health Organization [41].

We summarized descriptive data on household, maternal, and child characteristics from both survey rounds. Chi-square tests were used to assess differences between rounds according to the outcome and exposure variables. We described FS and FI levels according to selected predictor variables collected 12 and 18 months after birth. Odds ratios (OR), and their respective 95% Confidence Intervals (95%CI), of the association between FI and the selected predictors in each round, were estimated using crude and adjusted logistic regressions with robust variance. The final multivariable models were selected based on the Log-likelihood and the Akaike information criterion (AIC). The Variance Inflation Factor (VIF) test with a cut-off point of 10 was used to assess multicollinearity and determine variables’ permanence in the model.

For the food security and insecurity variation between 12 and 18 months, five categories were created by grouping the families according to their FI status on both survey rounds classified as: Food Secure—remained FS in both survey rounds; Became Food Secure—were FI at 12 month and FS at 18 months; Became Food Insecure—were FS at 12 month and FI at 18 months, Sustained Food Insecurity—mild and moderate FI on both rounds; and Sustained severe Food Insecurity—severe FI on both rounds. Chi-square tests were used to evaluate the association between FI variation and the exposure variables.

Statistical analyses were performed in STATA version 16.1 (StataCorp. 2019. Stata Statistical Software: Release 16. College Station, TX: StataCorp LP).

## Results

The analysis included data from 325 families followed up in round 2 and 331 families in round 3. FI was observed in 33.5% of the households in round 2, and in 42.9% in round 3. Severe FI, or hunger, was reported in 6.1% and 5.4% of the households in rounds 2 and 3, respectively. In 2022, an increase in the reported family’s monthly income was observed. While in 2021 40% of the sample earned one minimum wage (MW) or less, in 2022 this figure dropped to 24.5% (*p* < 0.001). The number of participants of cash transfer programs increased from 44.0% (2021) to 55.6% (2022) (*p* = 0.003) (Table 1). In 2021, 22.5% of the participants received the *Bolsa Familia* cash transfer (renamed as *Auxílio Brasil* on December 29, 2021 [6]) while 22.8% received the COVID-19 emergency cash transfer. In 2022, 52.6% of the families were beneficiaries of *Auxílio Brasil* and the COVID-19 emergency program had already ended (Data not shown). Other characteristics were similar between the two survey rounds (Table 1).

**Table 1:** Sample description according to household, maternal, and child characteristics 12 (*n* = 325) and 18 months (*n* = 331) after birth in a cohort of children born in Fortaleza during the COVID-19 pandemic

	12 months 2021	18 months 2022	*p*-value
	*n* (%)	*n* (%)	
*Family and household characteristics*			
Food security scale			0.022
Secure	109 (33.5)	142 (42.9)	
Mild insecurity	151 (46.4)	145 (43.8)	
Moderate insecurity	45 (14.0)	26 (7.9)	
Severe insecurity	20 (6.1)	18 (5.4)	
Number of residents			0.932
2–3	132 (40.6)	138 (41.7)	
4–5	152 (46.8)	154 (46.5)	
6 or more	41 (12.6)	39 (11.8)	
Number of residents under 18 years of age			0.974
1	138 (42.5)	139 (42.0)	
2	118 (36.3)	123 (37.1)	
3 or more	69 (21.2)	69 (20.9)	
Head of the household			0.974
Infant’s father	198 (60.9)	204 (61.6)	
Infant’s mother	83 (25.5)	82 (24.8)	
Other	44 (13.5)	45 (13.6)	
Education of the head of the household (years of formal education)			0.306
0–7 years	92 (28.4)	77 (23.3)	
8–11 years	207 (63.9)	229 (69.2)	
> 11 years	25 (7.7)	25 (7.6)	
Socioeconomic status^a^			0.929
A/B	24 (7.4)	22 (6.7)	
C1/C2	159 (48.9)	162 (48.9)	
D/E	142 (43.7)	147 (44.4)	
Family income (MW)^b^			< 0.001
Less than 1	130 (40.0)	81 (24.5)	
1–2	166 (51.1)	210 (63.4)	
3 or more	20 (8.9)	40 (12.1)	
Family income reduction after physical distancing begun			0.826
No	74 (22.8)	73 (22.1)	
Yes	251 (77.2)	258 (77.9)	
Participate in cash transfer programs			0.003
No	182 (56.0)	147 (44.4)	
Yes	143 (44.0)	184 (55.6)	
*Maternal characteristics*			
Skin color			0.925
White	59 (18.1)	57 (17.2)	
Brown	231 (71.1)	236 (71.3)	
Black	35 (10.8)	38 (11.5)	
Age			0.842
< 24 years	91 (28.0)	90 (27.2)	
25–29 years	94 (28.9)	91 (27.5)	
30–34 years	67 (20.6)	74 (22.4)	
≥ 35 years	73 (22.5)	76 (23.0)	
Education (years of formal education)			0.822
0–7 years	37 (11.5)	33 (10.0)	
8–11 years	221 (68.4)	229 (69.2)	
> 11 years	65 (20.1)	69 (20.9)	
Lives with a partner			0.754
No	81 (24.9)	86 (26.0)	
Yes	244 (75.1)	245 (74.0)	
Smoke			0.564
No	306 (94.1)	315 (95.2)	
Yes	19 (5.9)	16 (4.8)	
Alcohol consumption			0.219
No	264 (81.2)	256 (77.3)	
Yes	61 (18.8)	75 (22.7)	
Pre-pandemic working arrangements			0.986
Not working	129 (39.7)	132 (39.9)	
Informal	106 (32.6)	106 (32.0)	
Formal (CLT)^c^	90 (27.7)	93 (28.1)	
Continued working formally after March (physical distancing started)			0.992
Was not working	253 (72.1)	238 (71.9)	
Stopped working	24 (6.8)	23 (7.0)	
Continued working	74 (21.1)	70 (21.2)	
Maternal income reduction after physical distancing begun			0.853
No	126 (38.8)	126 (38.1)	
Yes	199 (61.2)	205 (61.9)	
Number of prenatal appointments			0.747
5 or less	45 (14.0)	43 (13.1)	
6 or more	277 (86.0)	285 (86.9)	
Common mental disorders			0.108
< 8	241 (74.1)	263 (79.5)	
≥ 8	84 (25.9)	68 (20.5)	
*Infant’s characteristics*			
Sex			0.894
Male	167 (51.4)	163 (51.9)	
Female	158 (48.6)	151 (48.1)	
Birth weight classification			0.995
Underweight	31 (9.5)	30 (9.6)	
Eutrophic	257 (79.1)	249 (79.3)	
Overweight	37 (11.4)	35 (11.1)	
Participates in visitation programs			0.203
No	304 (93.5)	317 (95.8)	
Yes	21 (6.5)	14 (4.2)	
Breastfeeding pattern			0.988
Bottle-feeding	99 (30.5)	99 (29.9)	
Complementary breastfeeding	153 (47.1)	157 (47.4)	
Predominant or exclusive breastfeeding	73 (22.5)	75 (22.7)	

^a^Stratified according to the Brazilian Socioeconomic Classification Criteria

^b^Brazilian Minimum Wage 2021 was $192

^c^SQR-20, Self-Report Questionnaire

Characteristics of the households facing FI were similar across survey rounds (Additional file 1: Table S2). Specifically, large households, with more children and adolescents, of low socioeconomic status, headed by women with lower education, that receive cash transfers, with mothers that presented CMD and with a history of smoking were associated with higher FI levels.

**Fig. 1:**
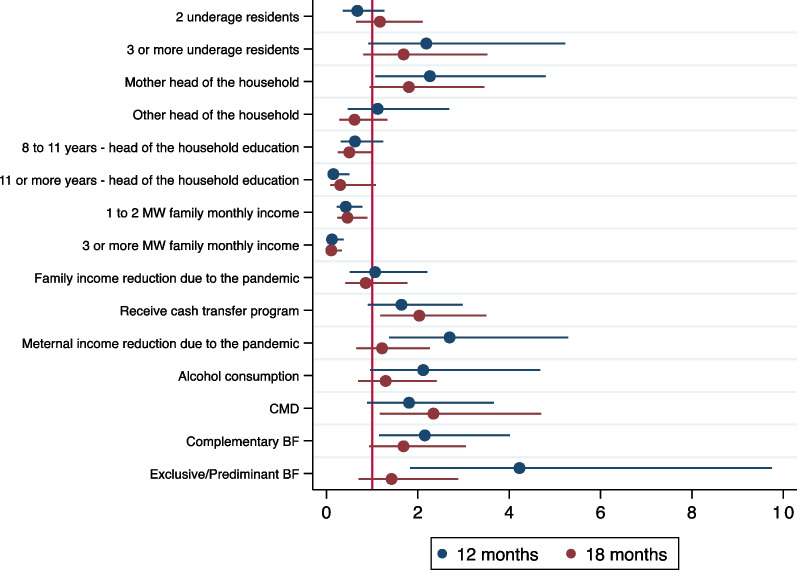
Multivariable association of FI, 12 and 18 months after birth, and potential risk factors in families with children born during the COVID-19 pandemic in Fortaleza, Ceará, Brazil. *Note* Variables displayed in the figure are those included in the final multivariable model (Additional file 1: Table S3). The reference groups are: 1 underage resident; father as the head of the household; under 8 years of formal education; less than 1 Minimum Wage (MW) of family monthly income; no family income reduction due to the pandemic; do not receive cash transfer programs; no maternal income reduction due to the pandemic; no alcohol consumption; no Common Mental Disorders (CMD); no breastfeeding (BF)

Figure 1 shows multivariable associations between FI and the selected predictors (Additional file 1: Table S3 shows the crude and adjusted OR and 95% CI). At 12-month follow-up, households whose heads had more years of formal education (8–11 years—OR 0.62, 95% CI 0.33; 1.18, and 12 or more years—OR: 0.15, 95% CI 0.05; 0.47) and higher family monthly income (1–2.9 minimum wages (MW)—OR 0.42, 95% CI 0.23; 0.76, and 3 or more MW—OR: 0.12, 95% CI 0.12; 0.37) had fewer odds of facing food insecurity compared to those in the least favored categories. Families that experienced a maternal income reduction due to the pandemic had almost three times higher odds of FI (OR 2.69; 95% CI 1.35; 5.40) compared to families with no income reduction. Complementary (OR 2.15, 95% CI 1.28; 3.93) and exclusive or predominantly breastfeeding (OR 4.22, 95% CI 1.81; 9.84) were also associated with higher odds of FI. Eighteen months after delivering a baby during the pandemic, families that received cash transfers (OR 2.17 95% CI 1.27; 3.70) had twice the chance of being in FI, while higher family income was associated to lower odds of FI (1–2.9 MW—OR 0.45, 95% CI 0.23; 0.88, and 3 or more MW—OR 0.09, 95% CI 0.03; 0.25).

Between the two survey rounds, 24% of the households with FS became food insecure (mild). Of those that had been classified with mild FI at 12 months, 34.9% improved their status, 52.7% remained in the same category, and 12.3% were at more severe FI at 18 months (8.2% with moderate and 4.1% with severe FI). For households that had moderate food insecurity, 77.3% improved their status with 68.2% going to mild FI and 9.1% becoming FS. Half of the households that were severely FI at the 12-month follow-up remained in that category at 18 months, none became FS, and 20% and 25% improved to moderate and mild FI, respectively (Table 2).

**Table 2:** Transitions in food insecurity between the second and third survey rounds of the Iracema-COVID cohort, Fortaleza, Ceará, Brazil

	Round 3				Total (%)
	None	Mild	Moderate	Severe	
*Round 2*					
None	**79 (76.0)**^**a**^	***25 (24.0)***^***c***^	***0.0 (0.0)***^***c***^	***0.0 (0.0)***^***c***^	104 (33.1)
Mild	*51 (34.9)*^*b*^	**77 (52.7)**^**a**^	***12 (8.2)***^***c***^	***6 (4.1)***^***c***^	146 (46.5)
Moderate	*4 (9.1)*^*b*^	*30 (68.2)*^*b*^	**9 (20.4)**^**a**^	***1 (2.3)***^***c***^	44 (14.0)
Severe	*0.0 (0.0)*^*b*^	*5 (25.0)*^*b*^	*4 (20.0)*^*b*^	**11 (55.0)**^**a**^	20 (6.4)
Total (%)	134 (42.7)	137 (43.6)	25 (8.0)	18 (5.7)	314 (100.0)

The table includes participants with information in both survey rounds (*n* = 314). Cells on the diagonal (bold font^a^) include households that remained in the same category of food insecurity in both survey rounds. Cells below the diagonal (italic font^b^) are households that improved in the scale of food insecurity, while those above the diagonal (bolditalic font^c^) became more food insecure from round 2–3

**Fig. 2:**
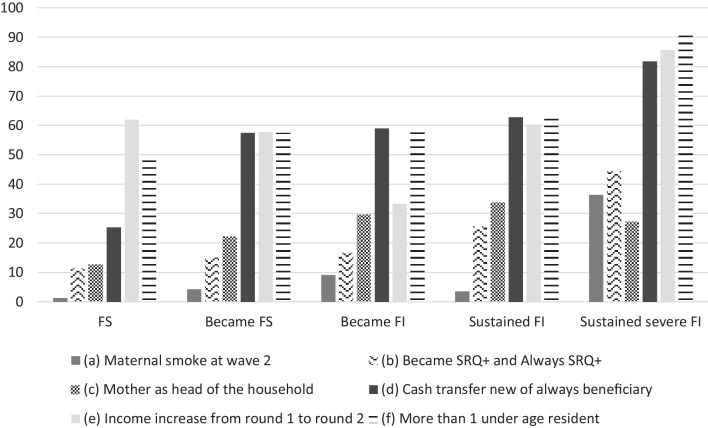
Food security and insecurity variation between the 12- and 18-months survey rounds according to predictor variables. Note: FS -Food secure, FI—Food insecure. SRQ + equals to Self-Report Questionnaire score of 8 or more points. *p*-values of the association of FI variation and the predictors between survey rounds: *a* < 0.001; *b* = 0.004; *c* = 0.004; *d* < 0.001; *e* = 0.005; *f* = 0.002

Household characteristics associated with sustained FI on both survey rounds include the head being the mother (*p* = 0.004), cash transfer benefits (*Bolsa Familia* or *Auxilio Brasil*) (*p* < 0.001), an increase in income between rounds (*p* = 0.050), and a larger number of children (*p* = 0.002)*.* Maternal characteristics associated with persistent FI include smoking (*p* < 0.001) and CMD (*p* = 0.004) (Fig. 2).

## Discussion

This study analyzed the prevalence of FI in a cohort of children born during the COVID-19 pandemic in Fortaleza who were followed up at 12 and 18 months after birth. At 12 months, we found 38 to 85% higher odds of FI in households whose head had fewer years of education and 2.3 times higher when it was a woman, had lower income (over 65%), whose mother faced income reduction during the pandemic (2.7 times), and practiced any type of breastfeeding (2.2 times higher when complementary and 4.2 times when had exclusively breastfed). At 18 months, factors that contributed to increase the odds of FI were lower family income, households that received cash transfer programs, and those whose mothers presented CMD. Households that faced FI at both survey rounds (*n* = 155) had more than one child or adolescent in the house, a higher prevalence of maternal smoking and maternal CMD, and received cash transfers (*Bolsa Familia* or *Auxilio Brasil).*

In 2013, 35.5% of the population in Ceará faced FI [18], increasing to almost half of the population (46.9%) in 2017–18 [22]. Recently, between 2017 and 2020, the state saw a 15% increase in FI [36]. Our results are consistent with that increasing trend.

The COVID-19 pandemic negatively affected the income and work conditions of the population in Brazil. In the Northeast, over 20% of the population reported having lost their jobs (20.4%), and over half had an income reduction (53.4%) due to the COVID-19 pandemic [35]. FI increase during the COVID-19 pandemic was observed globally; in 2020, moderate and severe FI had an increase equal to the previous 5 years combined and a noted increase in undernourishment [3]. In the United States, FI prevalence was 32.2% higher during the COVID-19 pandemic going from 18.8% before (in 2018) and 24.8% during (in 2020) the pandemic [32]. The effect of COVID-19 on FI levels could be associated with the disruption caused in food supply and availability due to unfavorable conditions for food production caused by the pandemic [40], but also with increased poverty due to disruption in the labor market.

Our findings show households headed by women have higher FI prevalence, even after adjusting for family income. Recent data collected in Brazil support our findings, with a higher prevalence of FI in households headed by women (11.2% and 19.3% in 2020 and 2021–22, respectively) compared to those headed by men (7.0% and 11.9% in 2020 and 2021–22, respectively) [34, 35]. Female-headed households are often associated with lower socioeconomic levels and single-parent households which would mean fewer income sources in the house [7].

In January 2019, when the 2019–2022 Brazilian government took term, the National Council for Food and Nutrition Security (CONSEA) was extinguished [9]. This drastically reduced efforts initiated in 2003 with the Zero Hunger program, which contributed to the decline in food insecurity that Brazil observed between 2000 and 2014. Although the conditional cash transfer was maintained, it was associated with higher FI in the 18-month follow-up. This is the opposite of a survey conducted in Ceará in 2007–8 that reported two times higher FS among families that received governmental aid [11]. Our results, however, reflect a scenario of severe social and sanitary consequences of COVID-19 that led to an increase in poverty. In Ceará it is estimated that 45.9% the population was living in poverty in 2021 (3.73 percentage points higher than in 2019) [31].

The increase in poverty was observed even though Brazil provided a COVID-19 emergency aid of about $112 (600 Brazilian Reais), launched in April, 2020. In 2020, 6.2% of households in Brazil lived exclusively on the COVID-19 emergency aid, and in low-income families the emergency aid covered 103% of the expected income had the pandemic not happened [8]. The emergency aid, however, ended in December 2020, and reinstated in April of 2021 with a reduced value of about $55 (300 Brazilian Reais), and ended in November. From January to April 2021, the worst moment of the COVID-19 pandemic in Brazil, no emergency aid was available.

In the 12-month survey round, 22.8% of the sample received the COVID-19 emergency aid, which represented 51.7% of all governmental transfer received by the participants of the Iracema-COVID. After the program’s closure, there was an increase in the proportion of *Bolsa Familia* beneficiaries (47.9% and 69.4% new recipients among the emergency aid non-beneficiaries and beneficiaries, respectively). Being in sustained severe FI was associated with receiving governmental cash transfers, and larger households with more underage residents. *Bolsa Familia* and the Emergency aid transfers to families (400 and 600 Brazilian *Reais*—about US$75 and US$ 112, respectively) have different purchasing power depending on families’ characteristics (e.g., female headed) and were possibly not enough to remove some families from a FI situation [24].

Compared to pre-pandemic data, there was an increase in the proportion of mothers that exclusively and predominantly breastfed their babies until the recommended 6 months of life [15]. Also, continuing to breastfeed the child after food introduction was positively associated with higher proportions of FI in our sample. These results suggest that mothers could have delayed solid food introduction and weaning considering the lower food availability in the households and thus assuring their infant would be fed and receive the minimum amount of energy and nutrients [16].

This study has some limitations. Our results are representative of a group of families with infants born during the COVID-19 pandemic in the fifth largest city in Brazil. They are representative of large urban areas in the country but cannot be generalized to other contexts.

## Conclusions

A decrease in FI was observed between 12 and 18 months after birth in families with children born during the COVID-19 pandemic. FI was more prevalent among households with lower socioeconomic level (income and education), and headed by mothers that became unemployed or suffered an income reduction during the COVID-19 pandemic. Although the prevalence of FI decreased in our sample, national data show that there is still a growing number of families with no regular access to enough and/or nutritionally appropriate food, being the Northeast region one of the most affected. We have identified the groups at higher FI risk that can be a reference to governmental policies.

## Supplementary Information

**Additional file 1.** Supplementary Tables.

## Data Availability

Data available upon request.
