# Supplementary material for: Food insecurity among families with infants born during the COVID-19 pandemic in Fortaleza, Northeast Brazil
Source: J Health Popul Nutr. 2023 Mar 5;42:14. doi: 10.1186/s41043-023-00354-w (PMC9985700; doi:10.1186/s41043-023-00354-w)
Supplement: Supplementary file 1 — Additional file 1. Supplementary Tables. [file 41043_2023_354_MOESM1_ESM.docx]

**Supplementary Table 1.** Description of exposure variables included in the study

| **INCLUDED VARIABLES** | **CATEGORIZATION** |
| --- | --- |
| **Household characteristics** |  |
| Number of residents | 2 to 3  4 to 5  6 or more |
| Number of residents under 18 years of age | 1  2  3 or more |
| Head of the family | Infant’s father  Infant’s mother  Other |
| Head of the family education level | 0 to 7 years  8 to 11 years  >11 years |
| Socioeconomic status (CCEB)*^a^ | A/B  C1/C2  D/E |
| Family current income (Brazilian Minimum Wage, ≈$190) | Less than 1  1 to 2  3 or more |
| Family income reduction after physical distancing  begun * | No  Yes |
| Government cash transfer program | No  Yes |
| **Maternal characteristics** |  |
| Skin color* | White  Brown  Black |
| Age (full years) | ≤ 24 years  25 to 29 years  30 to 34 years  ≥ 35 years |
| Living with a partner | No  Yes |
| Education level | 0 to 7 years  8 to 11 years  >11 years |
| Smoke | No  Yes |
| Alcohol consumption | No  Yes |
| Pre-pandemic working arrangements * | Not working  Informal  Formal (CLT)*** |
| Continued working formally (CLT) after March (physical distancing start) *^b^ | Was not working  Stopped working  Continued working |
| Maternal income reduction after physical distancing* | No  Yes |
| Number of prenatal appointments* | <5  ≥6 |
| Common mental disorders (SRQ-20) ^c^ | Negative (<8)  Positive (>7) |
| **Child’s characteristics** |  |
| Sex* | Male  Female |
| Birthweight classification | underweight <2500g  eutrophic 2500-3999g  overweight ≥4000g |
| Breastfeeding (BF) patterns* | Bottle-feeding  Complementary BF  Predominant and Exclusive BF |

*Collected during the Iracema-COVID cohort six months interview; ^a^Brazilian Economic Classification Criteria (*Critério de Classificação Econômica Brasil);* ^b^CLT – Brazilian consolidation of labor Law; ^c^SRQ-20 – Self-report Questionnaire

**Supplementary Table 2. Characteristics of the sample regarding food insecurity levels and maternal and infant factors in Fortaleza, Ceará, 12 and 18 months after birth in a cohort of children born during the COVID-19 pandemic.**

| **Characteristics** | **Iracema-COVID – 12 months (n=325)** | | | | | **Iracema-COVID – 18 months (n=331)** | | | | |
| --- | --- | --- | --- | --- | --- | --- | --- | --- | --- | --- |
|  | **Food**  **security (%)** | **Food Insecurity (%)** | | |  | **Food**  **security (%)** | **Food Insecurity (%)** | | |  |
|  |  | **Mild** | **Moderate** | **Severe** | **p-value** |  | **Mild** | **Moderate** | **Severe** | **p-value** |
| **Family and household Characteristics** | | | | | | | | | | |
| Number of residents |  |  |  |  | 0.001 |  |  |  |  | 0.033 |
| 2 to 3 | 36.4 | 49.2 | 13.6 | 0.8 |  | 50.7 | 37.7 | 9.4 | 2.2 |  |
| 4 to 5 | 33.5 | 45.4 | 14.5 | 6.6 |  | 39.0 | 48.1 | 5.8 | 7.1 |  |
| 6 or more | 24.4 | 41.5 | 12.2 | 22.0 |  | 30.7 | 48.7 | 10.3 | 10.3 |  |
| Number of residents <18 years |  |  |  |  | <0.001 |  |  |  |  | 0.001 |
| 1 | 38.4 | 45.7 | 13.0 | 2.9 |  | 52.5 | 36.7 | 9.4 | 1.4 |  |
| 2 | 38.1 | 49.2 | 11.0 | 1.7 |  | 39.8 | 49.6 | 5.7 | 4.9 |  |
| 3 or more | 15.9 | 43.5 | 20.3 | 20.3 |  | 28.9 | 47.8 | 8.7 | 14.5 |  |
| Head of the household |  |  |  |  | 0.026 |  |  |  |  | 0.045 |
| Infant’s father | 39.9 | 43.9 | 11.1 | 5.1 |  | 48.0 | 41.7 | 5.9 | 4.4 |  |
| Infant’s mother | 19.3 | 54.2 | 19.3 | 7.2 |  | 28.1 | 52.4 | 12.2 | 7.3 |  |
| Other | 31.8 | 43.2 | 15.9 | 9.1 |  | 46.7 | 37.8 | 8.9 | 6.7 |  |
| Education of the head of the household (years of formal education) | | |  |  | <0.001 |  |  |  |  | <0.001 |
| 0 to 7 years | 20.7 | 42.4 | 21.7 | 15.2 |  | 24.7 | 45.5 | 14.3 | 15.6 |  |
| 8 to 11 years | 34.8 | 50.2 | 12.1 | 2.9 |  | 45.4 | 45.9 | 6.1 | 2.6 |  |
| 12 or more years | 68.0 | 32.0 | 0.0 | 0.0 |  | 76.0 | 20.0 | 4.0 | 0.0 |  |
| Socioeconomic status^a^ |  |  |  |  | <0.001 |  |  |  |  | <0.001 |
| A/B | 70.8 | 25.0 | 4.2 | 0.0 |  | 63.6 | 31.8 | 4.6 | 0.0 |  |
| C1/C2 | 37.7 | 51.6 | 8.2 | 2.5 |  | 51.9 | 42.6 | 4.3 | 1.2 |  |
| D/E | 22.5 | 44.4 | 21.8 | 11.3 |  | 29.9 | 46.9 | 12.3 | 10.9 |  |
| Family income (MW)^b^ |  |  |  |  | <0.001 |  |  |  |  | <0.001 |
| Less than 1 | 17.7 | 52.9 | 19.6 | 9.8 |  | 17.7 | 52.3 | 21.5 | 8.5 |  |
| 1 to 2 | 34.7 | 47.4 | 13.2 | 4.7 |  | 39.2 | 45.8 | 10.2 | 4.8 |  |
| 3 or more | 75.8 | 21.2 | 0.0 | 3.0 |  | 72.4 | 24.1 | 0.0 | 3.5 |  |
| Family income reduction after physical distancing begun |  |  |  |  | 0.082 |  |  |  |  | 0.484 |
| No | 46.0 | 40.5 | 9.5 | 4.0 |  | 50.7 | 37.0 | 6.9 | 5.5 |  |
| Yes | 29.9 | 48.2 | 15.1 | 6.8 |  | 40.7 | 45.7 | 8.1 | 5.4 |  |
| Receive in cash transfer programs | |  |  |  | <0.001 |  |  |  |  | <0.001 |
| No | 42.3 | 42.9 | 12.6 | 2.2 |  | 59.9 | 32.7 | 5.4 | 2.0 |  |
| Yes | 22.4 | 51.0 | 15.4 | 11.2 |  | 28.9 | 52.8 | 10.0 | 8.3 |  |
| **Maternal Characteristics** | | | | | | | | | | |
| Skin color |  |  |  |  | 0.377 |  |  |  |  | 0.061 |
| White | 39.0 | 42.4 | 16.9 | 1.7 |  | 45.6 | 47.4 | 1.7 | 5.3 |  |
| Brown | 31.2 | 48.5 | 12.5 | 7.8 |  | 44.1 | 39.8 | 9.8 | 6.4 |  |
| Black | 40.0 | 40.0 | 17.1 | 2.9 |  | 31.6 | 63.2 | 5.3 | 0.0 |  |
| Age |  |  |  |  | 0.650 |  |  |  |  | 0.393 |
| 18 to 24 years | 29.7 | 46.1 | 16.5 | 7.7 |  | 41.1 | 42.2 | 11.1 | 5.6 |  |
| 25 to 29 years | 35.1 | 42.5 | 18.1 | 4.3 |  | 44.0 | 43.9 | 7.7 | 5.5 |  |
| 30 to 34 years | 34.3 | 52.2 | 9.0 | 4.5 |  | 50.0 | 40.5 | 8.1 | 1.4 |  |
| ≥ 35 years | 35.6 | 46.6 | 9.6 | 8.2 |  | 36.8 | 50.0 | 4.0 | 9.2 |  |
| Education (years of formal education) | |  |  |  | <0.001 |  |  |  |  | <0.001 |
| 0 to 7 years | 8.1 | 51.4 | 21.6 | 18.9 |  | 15.2 | 45.5 | 12.1 | 27.3 |  |
| 8 to 11 years | 31.2 | 47.5 | 15.4 | 5.9 |  | 42.4 | 45.4 | 8.3 | 3.9 |  |
| 12 or more years | 56.9 | 40.0 | 3.1 | 0.0 |  | 58.0 | 37.7 | 4.3 | 0.0 |  |
| Lives with a partner |  |  |  |  | 0.028 |  |  |  |  | 0.318 |
| No | 21.0 | 51.9 | 18.5 | 8.6 |  | 38.4 | 46.5 | 11.6 | 3.5 |  |
| Yes | 37.7 | 44.7 | 12.3 | 5.3 |  | 44.5 | 43.9 | 6.5 | 6.1 |  |
| Smoke |  |  |  |  | 0.002 |  |  |  |  | 0.001 |
| No | 35.0 | 46.7 | 13.4 | 4.9 |  | 44.1 | 44.1 | 7.6 | 4.1 |  |
| Yes | 10.5 | 42.1 | 21.1 | 26.3 |  | 18.8 | 37.5 | 12.5 | 31.3 |  |
| Alcohol consumption |  |  |  |  | 0.059 |  |  |  |  | 0.564 |
| No | 36.3 | 45.8 | 12.2 | 5.7 |  | 44.5 | 43.0 | 7.0 | 5.5 |  |
| Yes | 21.3 | 49.2 | 21.3 | 8.2 |  | 37.3 | 46.7 | 10.7 | 5.3 |  |
| Pre-pandemic working arrangements | |  |  |  | 0.030 |  |  |  |  | 0.025 |
| Not working | 29.5 | 46.5 | 14.7 | 9.3 |  | 37.1 | 46.2 | 6.1 | 10.6 |  |
| Informal | 27.4 | 50.0 | 17.9 | 4.7 |  | 43.4 | 43.4 | 10.4 | 2.8 |  |
| Formal (CLT)^c^ | 46.7 | 42.2 | 7.8 | 3.3 |  | 50.5 | 40.9 | 7.5 | 1.1 |  |
| Continued working formally after March (physical distancing start) |  |  |  |  | 0.011 |  |  |  |  | 0.091 |
| Was not working | 28.5 | 48.1 | 16.2 | 7.2 |  | 39.9 | 45.0 | 8.0 | 7.1 |  |
| Stopped working | 27.3 | 54.5 | 13.6 | 4.6 |  | 34.8 | 60.9 | 4.3 | 0.0 |  |
| Continued working | 53.0 | 38.2 | 5.9 | 2.9 |  | 55.7 | 34.3 | 8.6 | 1.4 |  |
| Maternal income reduction after physical distancing begun | | | |  | 0.018 |  |  |  |  | 0.055 |
| No | 43.7 | 39.7 | 10.3 | 6.3 |  | 47.6 | 37.3 | 6.4 | 8.7 |  |
| Yes | 27.1 | 50.8 | 16.1 | 6.0 |  | 40.0 | 47.8 | 8.8 | 3.4 |  |
| Number of prenatal appointments |  |  |  |  | 0.307 |  |  |  |  | 0.504 |
| 5 or less | 28.9 | 51.1 | 8.9 | 11.1 |  | 37.2 | 46.5 | 7.0 | 9.3 |  |
| 6 or more | 34.6 | 46.2 | 14.1 | 5.1 |  | 44.2 | 43.2 | 8.1 | 4.6 |  |
| Common Mental disorders (SRQ-20)^d^ | |  |  |  | <0.001 |  |  |  |  | <0.001 |
| <8 | 38.2 | 44.4 | 14.1 | 3.3 |  | 47.9 | 42.2 | 6.5 | 3.4 |  |
| ≥8 | 20.2 | 52.4 | 13.1 | 14.3 |  | 23.5 | 50.0 | 13.2 | 13.2 |  |
| **Infant’s characteristics** | | | | | | | | | | |
| Sex |  |  |  |  | 0.353 |  |  |  |  | 0.421 |
| Male | 35.3 | 46.7 | 10.8 | 7.2 |  | 42.9 | 45.4 | 5.5 | 6.1 |  |
| Female | 31.7 | 46.2 | 17.1 | 5.0 |  | 43.4 | 41.7 | 10.6 | 5.3 |  |
| Birth weight classification |  |  |  |  | 0.098 |  |  |  |  | 0.005 |
| Underweight | 29.0 | 58.1 | 9.7 | 3.2 |  | 56.7 | 33.3 | 10.0 | 0.0 |  |
| Eutrophic | 35.8 | 44.7 | 14.4 | 5.1 |  | 42.6 | 45.0 | 8.0 | 4.4 |  |
| Overweight | 21.6 | 48.7 | 13.5 | 16.2 |  | 31.4 | 42.9 | 5.7 | 20.0 |  |
| Participates in visitation programs |  |  |  |  | 0.565 |  |  |  |  | 0.818 |
| No | 34.2 | 46.4 | 13.5 | 5.9 |  | 42.9 | 43.5 | 7.9 | 5.7 |  |
| Yes | 23.8 | 47.6 | 19.1 | 9.5 |  | 42.9 | 50.0 | 7.1 | 0.0 |  |
| Breastfeeding pattern |  |  |  |  | 0.241 |  |  |  |  | 0.648 |
| Not breastfed | 42.4 | 43.4 | 9.1 | 5.1 |  | 48.5 | 40.4 | 7.1 | 4.0 |  |
| Complementary breastfeeding | 32.0 | 45.8 | 15.0 | 7.2 |  | 38.9 | 47.1 | 7.0 | 7.0 |  |
| Predominant or exclusive breastfeeding | 24.7 | 52.0 | 17.8 | 5.5 |  | 44.0 | 41.3 | 10.7 | 4.0 |  |
| p-value: Fisher Exact test; ^a^Stratified according to the Brazilian Socioeconomic Classification Criteria; ^b^Brazilian Minimum Wage 2021 - $192; ^c^CLT – Brazilian Consolidation of Labor Laws; ^d^Self-Report Questionnaire | | | | | | | | | | |

**Supplementary Table 3.** Crude and adjusted association of food insecurity 12 and 18 months after birth in families of children born during the COVID-19 pandemic and maternal and infant’s characteristics.

|  | **12 months (n=325)** | | **18 months (n=331)** | |
| --- | --- | --- | --- | --- |
|  | **Crude** | **Adjusted** | **Crude** | **Adjusted** |
|  | **OR (95%CI)** | **OR (95%CI)** | **OR (95%CI)** | **OR (95%CI)** |
| Family and household Characteristics | | | | |
| Number of residents | p=0.372 |  | p=0.036 |  |
| 2 to 3 | 1 |  | 1.61 (1.01; 2.57) |  |
| 4 to 5 | 1.13 (0.69; 1.85) |  | 2.32 (1.08; 4.95) |  |
| 6 or more | 1.77 (0.79; 3.93) |  |  |  |
| Number of residents under 18 years of age | p=0.004 | p=0.149 | p=0.043 | p=0.392 |
| 1 | 1 | 1 | 1 | 1 |
| 2 | 1.01 (0.61; 1.68) | 0.67 (0.36; 1.29) | 1.67 (1.02; 2.73) | 1.17 (0.65; 2.16) |
| 3 or more | 3.29 (1.58; 6.83) | 2.18 (0.89; 5.34) | 2.71 (1.46; 5.03) | 1.69 (0.80; 3.57) |
| Head of the family | p=0.005 | p=0.091 | p=0.009 | p=0.055 |
| Infant’s father | 1 | 1 | 1 | 1 |
| Infant’s mother | 2.78 (1.50; 5.15) | 2.26 (1.09; 4.72) | 2.37 (1.36; 4.13) | 1.80 (0.94; 3.47) |
| Other | 1.42 (0.71; 2.85) | 1.12 (0.46; 2.72) | 1.06 (0.55; 2.02) | 0.61 (0.29; 1.32) |
| Education of the head of the family (years of formal education) | p=0.001 | p=0.005 | p<0.001 | p=0.066 |
| 0 to 7 years | 1 | 1 | 1 | 1 |
| 8 to 11 years | 0.49 (0.27; 0.87) | 0.62 (0.33; 1.18) | 0.39 (0.22; 0.70) | 0.50 (0.25; 0.98) |
| 12 or more years | 0.12 (0.05; 0.33) | 0.15 (0.05; 0.47) | 0.10 (0.04; 0.30) | 0.30 (0.09; 0.99) |
| Socioeconomic status  (Critério Brasil – ABEP,  2021)^a^ | p<0.001 |  | p<0.001 |  |
| A/B | 1 |  | 1 |  |
| C1/C2 | 4.01 (1.57; 10.24) |  | 1.63 (0.65; 4.09) |  |
| D/E | 8.35 (3.18; 21.93) |  | 4.10 (1.60; 10.48) |  |
| Family income (Minimum Wage) | p<0.001 | p<0.001 | p<0.001 | p<0.001 |
| Less than 1 | 1 | 1 | 1 | 1 |
| 1 to 2.9 | 0.33 (0.19; 0.58) | 0.42 (0.23; 0.76) | 0.35 (0.19; 0.63) | 0.46 (0.23; 0.92) |
| 3 or more | 0.08 (0.03; 0.21) | 0.12 (0.04; 0.37) | 0.05 (0.02; 0.13) | 0.11 (0.03; 0.34) |
| Family income reduction  after physical distancing  begun | p=0.011 | p=0.875 | p=0.130 | p=0.676 |
| No | 1 | 1 | 1 | 1 |
| Yes | 1.99 (1.17; 3.40) | 1.06 (0.49; 2.31) | 1.50 (0.89; 2.53) | 0.86 (0.42; 1.75) |
| Participate in cash transfer programs | p<0.001 | p=0.111 | p<0.001 | p=0.013 |
| No | 1 | 1 | 1 | 1 |
| Yes | 2.54 (1.56; 4.16) | 1.64 (0.89; 3.02) | 3.67 (2.31; 5.82) | 2.03 (1.17; 3.55) |
| Maternal Characteristic | | | | |
| Skin color | p=0.367 |  | p=0.326 |  |
| White | 1 |  | 1 |  |
| Brown | 1.41 (0.78; 2.55) |  | 1.06 (0.59; 1.91) |  |
| Black | 0.96 (0.41; 2.25) |  | 1.82 (0.77; 4.30) |  |
| Age | p=0.833 |  | p=0.728 |  |
| 18 to 24 years | 1.31 (0.68; 2.53) |  | 0.92 (0.48; 1.75) |  |
| 25 to 29 years | 1.02 (0.54; 1.94) |  | 0.86 (0.45; 1.62) |  |
| 30 to 34 years | 1.06 (0.53; 2.12) |  | 0.68 (0.34; 1.36) |  |
| ≥ 35 years | 1 |  | 1 |  |
| Education (years of formal education) | p<0.001 |  | p=0.001 |  |
| 0 to 7 years | 1 |  | 1 |  |
| 8 to 11 years | 0.19 (0.06; 0.66) |  | 0.24 (0.09; 0.65) |  |
| 12 or more years | 0.07 (0.02; 0.24) |  | 0.13 (0.04; 0.38) |  |
| Lives with a partner | p=0.007 |  | p=0.325 |  |
| No | 1 |  | 1 |  |
| Yes | 0.44 (0.24; 0.80) |  | 0.78 (0.47; 1.28) |  |
| Pre-pandemic working arrangements | p=0.010 |  | p=0.136 |  |
| Not working | 1 |  | 1 |  |
| Informal | 1.11 (0.63; 1.96) |  | 0.77 (0.46; 1.30) |  |
| Formal (CLT)^b^ | 0.48 (0.27; 0.84) |  | 0.58 (0.34; 0.99) |  |
| Continued working  formally (CLT) after  March (physical distancing  start) | p=0.001 |  | p=0.049 |  |
| Was not working | 1 |  | 1 |  |
| Stopped working | 1.06 (0.40; 2.83) |  | 1.25 (0.51; 3.06) |  |
| Continued working | 0.35 (0.20; 0.62) |  | 0.53 (0.31; 0.91) |  |
| Maternal income reduction  after physical distancing  begun | p=0.002 | p=0.005 | p=0.175 | p=0.534 |
| No | 1 | 1 | 1 | 1 |
| Yes | 2.08 (1.30; 3.33) | 2.69 (1.35; 5.40) | 1.36 (0.87; 2.13) | 0.86 (0.42; 1.75) |
| Smoke | p=0.045 |  | p=0.059 |  |
| No | 1 |  | 1 |  |
| Yes | 4.57 (1.03; 20.20) |  | 3.42 (0.95; 12.27) |  |
| Alcohol consumption | p=0.027 | p=0.074 | p=0.270 | p=0.413 |
| No | 1 | 1 | 1 | 1 |
| Yes | 2.11 (1.09; 4.10) | 2.12 (0.93; 4.83) | 1.35 (0.79; 2.29) | 1.30 (0.70; 2.41) |
| Number of prenatal appointments | p=0.450 |  | p=0.390 |  |
| 5 or less | 1.30 (0.65; 2.61) |  | 1 |  |
| 6 or more | 1 |  | 1.34 (0.69; 2.59) |  |
| Common Mental Disorders (SRQ-20)^c^ | p=0.003 | p=0.086 | p<0.001 | p=0.009 |
| <8 | 1 | 1 | 1 | 1 |
| ≥8 | 2.43 (1.34; 4.40) | 1.81 (0.92; 3.55) | 2.99 (1.62; 5.51) | 2.34 (1.24; 4.42) |
| Infant’s characteristics | | | | |
| Sex | p=0.483 |  | p=0.920 |  |
| Male | 1 |  | 1 |  |
| Female | 1.18 (0.74; 1.87) |  | 1.02 (0.65; 1.60) |  |
| Birth weight classification | p=0.208 |  | p=0.129 |  |
| Underweight | 1 |  | 1 |  |
| Eutrophic | 0.73 (0.32; 1.66) |  | 1.76 (0.82; 3.79) |  |
| Overweight | 1.48 (0.49; 4.47) |  | 2.85 (1.03; 7.89) |  |
| Participates in visitation program | p=0.334 |  | p=0.997 |  |
| No | 1 |  | 1 |  |
| Yes | 1.66 (0.59; 4.68) |  | 1.00 (0.34; 2.96) |  |
| Breastfeeding pattern | p=0.047 | p=0.020 | p=0.312 | p=0.225 |
| Not breastfed | 1 | 1 | 1 | 1 |
| Complementary breastfeeding | 1.56 (0.93; 2.64) | 2.15 (1.28; 3.93) | 1.48 (0.89; 2.46) | 1.69 (0.93; 3.07) |
| Predominant or exclusive breastfeeding | 2.25 (1.16; 4.38) | 4.22 (1.81; 9.84) |  | 1.43 (0.72; 2.83) |

*^a^*Stratified according to the Brazilian Socioeconomic Classification Criteria; ^b^CLT – Brazilian Consolidation of Labor Laws; ^c^Self-Report Questionnaire; †Brazilian Minimum Wage 2021 - $192
